# A Federated Learning Model Based on Hardware Acceleration for the Early Detection of Alzheimer’s Disease

**DOI:** 10.3390/s23198272

**Published:** 2023-10-06

**Authors:** Kasem Khalil, Mohammad Mahbubur Rahman Khan Mamun, Ahmed Sherif, Mohamed Said Elsersy, Ahmad Abdel-Aliem Imam, Mohamed Mahmoud, Maazen Alsabaan

**Affiliations:** 1 Electrical and Computer Engineering Department, University of Mississippi, Oxford, MS 38677, USA; 2 Department of Electrical Engineering, Assiut University, Assiut 71515, Egypt; 3 Electrical and Computer Engineering Department, Tennessee Technological University, Cookeville, TN 38505, USA; mrkhanmamu42@tntech.edu (M.M.R.K.M.); mmahmoud@tntech.edu (M.M.); 4 School of Computing Sciences and Computer Engineering, University of Southern Mississippi, Hattiesburg, MS 39406, USA; 5 Computer Information Systems Department, Higher Colleges of Technology, Al Ain 25026, United Arab Emirates; melsersy@hct.ac.ae; 6 College of Osteopathic Medicine, William Carey University, Hattiesburg, MS 39401, USA; aimam@wmcarey.edu; 7 Department of Computer Engineering, College of Computer and Information Sciences, King Saud University, Riyadh 11362, Saudi Arabia; malsabaan@ksu.edu.sa

**Keywords:** Alzheimer’s disease, hardware acceleration, blood bio-samples, federated learning, early diagnosis

## Abstract

Alzheimer’s disease (AD) is a progressive illness with a slow start that lasts many years; the disease’s consequences are devastating to the patient and the patient’s family. If detected early, the disease’s impact and prognosis can be altered significantly. Blood biosamples are often employed in simple medical testing since they are cost-effective and easy to collect and analyze. This research provides a diagnostic model for Alzheimer’s disease based on federated learning (FL) and hardware acceleration using blood biosamples. We used blood biosample datasets provided by the ADNI website to compare and evaluate the performance of our models. FL has been used to train a shared model without sharing local devices’ raw data with a central server to preserve privacy. We developed a hardware acceleration approach for building our FL model so that we could speed up the training and testing procedures. The VHDL hardware description language and an Altera 10 GX FPGA are utilized to construct the hardware-accelerator approach. The results of the simulations reveal that the proposed methods achieve accuracy and sensitivity for early detection of 89% and 87%, respectively, while simultaneously requiring less time to train than other algorithms considered to be state-of-the-art. The proposed algorithms have a power consumption ranging from 35 to 39 mW, which qualifies them for use in limited devices. Furthermore, the result shows that the proposed method has a lower inference latency (61 ms) than the existing methods with fewer resources.

## 1. Introduction

Diagnosing mental health diseases is challenging due to the underlying conditions that lead to mental illness because of the subjective nature of the associated symptoms. Alzheimer’s disease (AD) is an example of this type of illness [1,2]. The avoidance of AD should be a primary focus of public health efforts. Approximately 6.5 million people in the United States suffer from this disease, bringing the total number of affected individuals worldwide to 55 million, over 60% of whom live in low-and middle-income countries. Every year, there are nearly 10 million new cases [3,4]. The elderly already have a high prevalence of AD, but it is anticipated to increase as life expectancy increases. The rate of AD, which is currently relatively high, is expected to rise further due to these developments and the falling birth rate. AD is the most prevalent form of dementia; nevertheless, despite the expenditure of billions of dollars on research, there are no drugs available that can treat the disease at this time [5].

The only way to lessen the impact of a public health catastrophe of this magnitude is to take preventative measures [6]. AD has a significant impact on a person’s day-to-day activities and routines. Patients with Alzheimer’s may exhibit behavioral changes, challenges related to their personality, and other clinical implications. Recent research has made significant efforts to describe the complicated interplay of genetic, environmental, and lifestyle variables in AD development. These strides have opened up new pathways for prospective medicines to combat this awful scenario [7]. Even though there is currently no treatment that can reverse the effects of degenerative dementia, early diagnosis and treatment can help reduce symptoms and delay the start of the condition. Additionally, it is believed that 23% of the causes of dementia are reversible in the early stages of the disease. The initial symptoms of dementia are often a deterioration in an individual’s capacity to speak or think critically.

A thorough examination of a patient’s medical history, behavioral analysis, cognitive testing, brain imaging, and blood collection are all necessary steps in the diagnostic process for AD. The patient’s medical history and a study of the patient’s behavior are essential pieces of information that are necessary in order to make a clinical diagnosis. However, detailed medical histories are required to conduct thorough behavioral and medical assessments over an extended period of time and during several visits from professionals. It is essential to set up a diagnostic strategy that can be accepted. Positron emission tomography (PET) and magnetic resonance imaging (MRI) are two examples of imaging techniques that can provide more convincing evidence to support a diagnosis of AD. Researchers have developed several valuable algorithms that may be used with a wide variety of data sources to diagnose AD based on medical imaging [8]. Biomarkers tau and amyloid beta (Aβ) are measured via PET and lumbar puncture (LP) to acquire cerebrospinal fluid (CSF) [9]. These pathological cornerstones are currently valuable markers for AD diagnosis and have helped clarify the disease’s biological characterization. Currently, measurements of these indicators can be performed in vivo [10,11]. PET imaging and lumbar puncture are both excessively disruptive methods of gathering CSE and are costly to use regularly in research or primary care, which is where the majority of dementia diagnoses are made. On the other hand, the rapid development of mass spectrometry and sensitive immunoassay techniques has made it possible to quickly uncover and validate plasma biomarkers for AD pathology.

Blood biomarkers are anticipated to improve the early diagnosis of persons with dementia and cognitive symptoms [12]. Plasma biomarkers will make it easier to research the mechanisms underlying diseases, resulting in improved symptom treatment and higher overall healthcare quality for patients. Recent research inquiries have uncovered an association between blood levels and Aβ deposition [13,14], astrogliosis [15], and neurodegeneration [16,17].One mechanism that may explain AD incidence is that, through chemotactic signaling, astrocytes move toward areas of neural lesions and insults. Upon interacting with immobilized beta-amyloid peptides, astrocytes stop their movement towards the sites of neural injury, and the astroglial clearance of beta-amyloid peptides decreases, which may be a factor in the pathology behind AD [18]. Tau phosphorylated at threonine 181 may currently be the blood biomarker for AD with the most clinical significance. Its excellent specificity for AD, strong associations with tau PET and Aβ, and high precision for predicting the development of AD dementia, according to several studies conducted on independent cohorts, are shown in [19]. The presence of beta-amyloid protein expresses the progression of AD. In [20], plasma Aβ42/Aβ40 showed a high correlation with the status of beta-amyloid protein in the brain. Diagnosing AD significantly by analyzing blood samples with artificial intelligence (AI) is now possible. This can happen years before apparent symptoms arise. This paper presents a hardware-based AI model for determining the likelihood of developing AD based on examining blood samples. By training a classification algorithm on the levels of tau and (Aβ) protein in a blood sample, it is feasible to differentiate between a healthy individual and one with a cognitive problem (such as mild cognitive impairment (MCI) or dementia). Because the hardware-based AI model can analyze vast volumes of data and correctly categorize it, this method can improve the overall accuracy of AD screening. In order to train and evaluate the models, blood samples from patients diagnosed with AD were used. The dataset classifies individuals of varying ages according to whether or not they have AD. The information in the dataset comes from a wide range of persons, both in terms of gender and health status.

In federated learning (FL), several devices, such as edge devices, train a single model without sending raw data to a centralized server. This is accomplished by collaborating on the training process. People in the early stages of AD are great candidates for FL, since it has a high success rate and can preserve the privacy of patients’ data. The FL can develop a local model with the help of the given data, which it may subsequently change to achieve maximum precision with the fewest possible losses. The VHDL hardware description language and an Altera 10 GX FPGA are utilized to construct the hardware-accelerator approach for our FL model. It is also implemented on a Synopsys Design compiler using 45 nm technology. The results of our performance evaluation indicate that the proposed approaches provide higher accuracy (89%) and sensitivity (87%) for early detection than the state-of-the-art algorithms, all while taking a shorter time for training. In addition, the proposed algorithms consume between 35 and 39 mW of power, making them an excellent choice for devices with limited resources. The FL method has an inference latency of 61 ms, which is lower than the traditional methods.

In summary, this paper provides the following contributions:

- The performances of the conventional machine learning algorithms were compared using the ADNI dataset to find the suitable one to use in the FL technique.
- The impact of the implementation of the FL technique to diagnose Alzheimer’s disease was analyzed.
- Proposing hardware accelerator with low power consumption and acceptable resource utilization for our FL model.

The remaining sections of our paper are organized as follows: In Section 2, we briefly review other existing schemes. In Section 3, we explain our proposed scheme. Next, we delve into the details of our proposed scheme implementation and performance evaluation in Section 4. This paper then concludes in Section 5.

## 2. Literature Review

A dementia prediction system for monitoring physical activity in the elderly was introduced in [21]. The principal component analysis (PCA) algorithm is used on data collected from infrared sensors to extract the main characteristics. A deep neural network is employed to classify the retrieved characteristics and predict the likelihood of dementia. The approach is used with 18 people with varying daily living situations. The approach has a prediction accuracy of 63.38%. An MRI-based dementia detection approach was introduced in [22]. MRI images are integrated with deep convolutional generative adversarial networks (GANs) to diagnose and categorize various kinds of dementia. The detection process includes the identification of early-onset dementia or MCI. For MCI prediction, the technique has a 74% accuracy. With the help of brain MRI data from the AD Neuroimaging Initiative stages, multiple works [23,24,25,26] investigate the application of FL to detect AD early. In [23], the authors investigate the application of federated deep learning for AD identification. The researchers created a FL architecture to solve privacy issues and problems with centralizing massive datasets. They achieved great performance in AD classification using data from three separately acquired ADNI stages to train a 3D convolutional neural network (CNN). The FL model using pre-trained weights produced the best results. The work addresses concerns about data decentralization and privacy while highlighting the promise of FL in neuroimaging for high-performance AD prediction.

Convolutional neural networks (CNN) were used by Arifoglu et al. [27] to determine whether or not a person with dementia behaves in a manner that has repercussions. To represent the behavioral challenges of those affected by dementia, synthetic data are developed. CNN is used to create activity sequence patterns that may be labeled. It learns to recognize unusual behavior caused by dementia by observing alterations in typical patterns. A dementia categorization technique was presented in [28] by Hanai et al. This technique is based on spontaneous speech analysis. To identify dementia, the approach employs informal conversation during a clinical interview. It finds and classifies dementia by using voice characteristics to answer questions. The approach was used on 136 individuals aged 45 to 84 to encompass Alzheimer’s and frontotemporal lobar degeneration (FTLD), with a classification accuracy of 77%.

In [29], the researchers employ three different neuroanatomical computational approaches, specifically 3D-Subject, 3D-Patches, and 3D-Slices, to develop a deep learning model capable of multimodal multi-class classification for Alzheimer’s disease. The model utilizes T1w-MRI and AV-45 PET scans acquired from the ADNI database for both three-class and two-class classifications of Alzheimer’s disease. Moreover, the study implemented a patch-extraction algorithm utilizing the torch package to introduce patches of different sizes. Consequently, this approach created distinct datasets consisting of patch sizes ranging from 32 to 88. In [30], the authors employed a structural biomarker known as structural magnetic resonance imaging to examine the alterations in neurostructure within various brain regions among individuals with AD, mild cognitive impairment (MCI), and those with normal cognitive function. The study employed conventional machine learning and ensemble learning models to identify AD and its various subtypes.

Hardware acceleration significantly impacts disease detection techniques in several ways, providing faster processing, improved accuracy, deployment of real-time applications, scalability, and edge computing. An FPGA accelerator is proposed for an AI-based analysis with an electrocardiogram signal to monitor the heart condition [31]. A one-dimensional convolutional neural network has been implemented using the hardware accelerator. It has the functionality of pipelined transformations, threshold calculation, and heartbeat count without multiplexed usage (mutual dependencies) of hardware resources on FPGA. The accelerator achieves higher speed compared to software implementation for portable ECG monitoring. In another study, to reduce the computational overhead and hardware complexity, a new algorithm named the coordinated rotation digital computer algorithm was implemented [32]. An FPGA was used as a controller and signal processor for transmitting, storing, processing, data acquisition, and finally displaying the signal. In [33], the authors used a hardware accelerator implemented with a sample prototype of an AI-based Flask-driven web application to predict diseases based on X-ray images. The authors found that FPGA hardware is advantageous over GPUs considering deep learning modules and attained fewer gate counts and lower power consumption.

A heart sound classification algorithm was implemented using CNN on a small-scale SoC-FPGA with fewer resources in [34]. FPGA with the parallelism of CNN and several techniques, such as loop unrolling, a fixed point of the model parameter, and reducing global memory, were applied to accelerate the algorithm. The result showed the classification speed improved by 3.13 times compared to CPU use. An embedded hardware-based cell classifier performed with nearly 100% accuracy while detecting kidney cell damage in [35]. The researchers proposed a real-time framework to detect cell toxicity using a shallow neural network on an FPGA device. Compared to software where the image detection takes 220 ms, the FPGA device requires only 400 ns to compute one sample.

A diabetic retinopathy (DR) diagnosis system using an FPGA was implemented in [36]. After quantization, CNN was trained on GPUs and deployed on FPGA, with an average processing time of 16.92 ms over CPU and 0.21 ms over GPU. The choice of FPGA was justified to provide a fast DR diagnosis for an effective intervention plan. Some other disease diagnosis techniques implemented in recent years involved the use of hardware accelerators that achieve faster performance and less power consumption, such as a CNN-based cough detection system on lightweight FGPA [37], FPGA-based real-time detection of Gait freezing with Parkinson patients [38] and epileptic seizure detection with wearable devices while implementing FPGA [39].

More specifically, due to the advantages of the FPGA accelerator, recently, the diagnosis of Alzheimer’s has reached a new pace. Possible use of Hardware acceleration through specialized GPUs, efficient implementation of complex machine learning algorithms, more rapid processing and analysis of MRI images, and deployment of real-time processing on IoT devices or in remote care are some advantages that can be utilized. In [40], the authors proposed a scheme for assisting Alzheimer’s patients in the moderate stage using smart glass to identify the patient. The technique was implemented on a Raspberry Pi 4 camera, an ultrasonic sensor, and a GPS module using several machine-learning algorithms. By monitoring daily activity to detect when skills are deteriorating, an IoT system based on a wearable prototype was implemented for potential Alzheimer’s patients in remote locations [41]. The data captured by the device can detect abnormal changes in daily routine to monitor disease progression.

Several other methods of detecting AD using image data, such as MRI images other than blood biomarkers, were used in this study. To the best of our knowledge, we are the first to offer the use of blood biomarkers with the FL in early AD detection. We refrain from comparing our method, which employs plasma biomarkers to identify Alzheimer’s, with studies that employ MRI data. These techniques work with different kinds of data, and the variables that affect them can affect how effective they are. Because of this, direct comparisons might not offer a fair or accurate evaluation of their unique qualities and prospective contributions. Unlike the previous work, our proposed work employs blood sample data to ensure AD detection is achieved with lightweight resources and in a faster manner. Furthermore, the hardware implementation of our proposed method has lower resource utilization, power consumption, and latency than the traditional methods mentioned earlier.

## 3. The Proposed Methods

In the following subsections, we will explain our proposed method.

### 3.1. Federated Learning

Federated learning (FL) is a type of ML that allows multiple devices or servers to work together without transferring data among each other. The central server, which coordinates the whole process, trains the final model in a decentralized manner. FL significantly differs from conventional ML in terms of data ownership, data privacy, scalability, and robustness. In FL, data remain on participant devices, and the central server coordinates and oversees the training process without accessing the data. In addition, FL works in cases where data are spread out on many devices, and privacy is a significant concern. In particular, real-time monitoring while providing security and privacy to personal data is now a real possibility due to the use of FL [42]. FL can reduce the cost of communication and latency associated with data transfer to a central server. Currently, FL is an active area of research aiming to improve FL’s efficiency, applicability, and scalability. In edge computing, researchers are trying to train models using data from edge devices to help reduce communication costs and preserve privacy by keeping the data on edge devices [43].

There are studies in security and privacy using FL, such as differential privacy, secure multi-party computation, secure aggregation, etc. [44]. In natural language processing (NLP) tasks such as language modeling and sentiment analysis, the involvement of FL may improve privacy with sensitive data such as financial or healthcare information. Additionally, in deep reinforcement learning in areas such as robotics and gaming, the application of FL can address the challenges of scalability and privacy with decentralized data [45]. More specifically, with healthcare data, FL has great potential to address data privacy challenges. FL can be used to train predictive models in which local model parameters will come from different organizations. Additionally, FL can be used to diagnose patients whose data will reside at respective locations or hospitals, but the data’s impact will pass to the main hospital through a model. Different aspects of drug discovery, such as checking the efficacy and safety of a new drug, can be studied using data from multiple sources without sharing these data.

FL involves multiple devices collaborating without sharing data, while model parameters are sent to the central server to make the model more robust. Initially, the central server coordinates among the devices involved in the model preparation process to initialize. After that, each device uses its respective data to prepare a model based on initial seed information from a central server. The central server receives model updates or parameters from the participating devices and combines them to create a central model. The combined model is sent back to the devices, and the process continues until the global central model converges [42]. FL has several advantages; for example, it can expand or scale into a larger dataset by expanding or distributing among a more significant number of machines or devices, improving the model’s robustness and generalization. On the other hand, due to being distributed in nature, while some of the local machines or devices become unavailable, the central model remains active and runs based on other distributed active devices.

Compared to the conventional ML algorithms, one distinct feature of FL is the aggregation of the model parameters produced by local devices via the central server. Several parameters aggregation techniques are available, such as federated averaging, federated stochastic gradient descent, and FL using momentum. The most common approach is the one with a federated average, where the weighted average of the model parameters from all devices are aggregated, and the weights are based on the size of the local device’s dataset. So, the larger dataset contributes heavily to the global model preparation compared to smaller local devices with a smaller dataset. The coordination process between the central server and the locally distributed devices or machines is highly significant because it dictates the balance between the model accuracy and communication efficiency. The central server must preserve the privacy of each of the local devices’ data and maintain the accuracy of the global model. The responsibilities of the central server include (but are not limited to) choosing the aggregation method, communication protocol, and criteria for convergence. The central server monitors the training process to make sure the process runs efficiently.

FL presents a potent paradigm for collaborative model creation in the context of detecting AD. FL enables collaboration across several medical facilities or research institutes to develop reliable diagnosis models without disclosing private patient information. In accordance with stringent healthcare rules like HIPAA and GDPR, this strategy guarantees that patient data stay private and secure. Because FL is decentralized, local devices or servers can update models based on their own data, which are then combined by a central server to produce a global model. The numerous and dispersed data sources help this global model, which might result in more precise and early identification of AD. In terms of detecting AD, FL has several privacy-related advantages, one of which is that it makes it possible to protect the privacy of specific patient data. Only model updates or gradients are exchanged with the central server when using FL; patient data are kept on local devices or servers. This eliminates the possibility of data breaches or unauthorized access by ensuring that critical patient information never leaves its source. Furthermore, methods like secure aggregation procedures and encryption may be used to further safeguard the confidentiality of patient data during the model-training procedure. Overall, FL offers a solid method for creating diagnostic models while maintaining the strictest privacy regulations for patient data.

Although FL provides privacy while combining ML models from distributed devices, it also comes with challenges or limitations. The distributed devices can be heterogeneous; they may differ in storage, computation power, and communication abilities. Thus, the central server needs to be efficient enough in coordination. Another issue is the statistical heterogeneity of devices, where identically distributed data in different devices may create confusion. Although sharing only model parameters is a good step toward data privacy, communicating model updates can still reveal sensitive information. Recently, secure multi-party computation and differential privacy aim to enhance privacy, but these processes come with the disadvantage of reduced efficiency and performance in models [44].

### 3.2. Dataset Description

The dataset for this paper was acquired from the ADNI website [46] after maintaining the necessary procedure of registration and approval. It contains different blood parameters and markers from high-precision mass spectrometry assay. Plasma samples were collected with normal cognitive conditions within one and a half years following an amyloid PET scan. The dataset contained 73 columns, including experimental procedures and different biomarkers. Based on the strong correlation with dementia, only 4 features out of 73 were selected to be used in our algorithms [20]. Those are peak areas of αβ40 and αβ42 with N-14 and N-15 isotopes. The total number of samples is 622. The experiment used two different mass spectrometer instruments: Lomus and Altis. Thermo Fisher Scientific develops both of them, and Lumos is a part of the Orbitrap series of mass spectrometers, which are well known for their accuracy and high-resolution abilities. Furthermore, Altis is part of triple quadrupole mass spectrometers widely used in chemical analysis with high-performance capabilities.

### 3.3. Data Preprocessing

The preprocessing step is an important step that ensures that proper data cleaning, transformation, and reduction have been accomplished. Data cleaning involves identifying and removing or correcting the data that contain errors, missing values, or other types of inconsistencies, improving the dataset’s quality. In data integration, when data come from different sources, there must be an action plan for correctly combining or merging the data. In the data transformation step, the data need to be transformed to fit the assumptions of a statistical model to help train machine learning (ML) models. Common transformation techniques include standardization, scaling, normalization, etc. Data reduction and feature selection are the two most essential data preprocessing steps, in which the use of sampling or dimensionality reduction is performed for large datasets to comply with the data reduction step and relevant but nonredundant features are selected using different methods in the feature selection step. The specific steps required depend on the type of dataset, the objective, and the input type expected to train and test the model.

Initially, the dataset was checked for any duplicate or missing data. The data column which indicates whether the participant with the sample has Alzheimer’s or not was given in numeric form. Since this study is expected to produce classification, the target variable was transformed from a numerical variable to a categorical variable (positive or negative). Then, the distribution of the features concerning each other was analyzed to check whether the chosen features were relevant. In addition, any outlier or unexpected spike in value was checked.

Finally, the data were normalized to improve model performance, converge faster, and avoid overfitting. Normalization makes the data scale-independent and ensures no particular feature dominates based on a larger amplitude. This way, it prevents bias. In particular, it is important for distance-based algorithms, which are specifically sensitive to the scale of input values. Regarding outliers, the data normalization brings those values closer to the mean, reducing their influence. In this paper, the ’MinMaxScaler’ function has been used to normalize the selected features of the dataset to keep the numerical data within 0–1. The formula subtracts the minimum value of specific features from each data point from that column and divides them by the range of the original data in that column. Then, the result has been scaled to the desired range (here, 0 to 1).

### 3.4. Model Preparation

**Figure 1:**
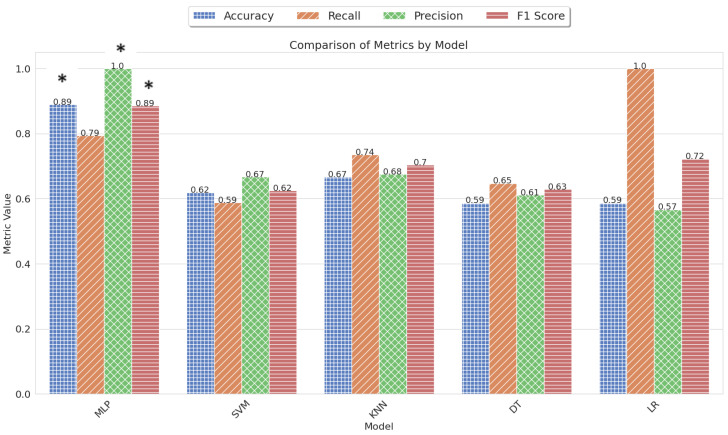
Comparison of performance matrices among models.

After dividing the data into training (90%) and testing (10%) segments, the processed data were input for five different classifier algorithms. These algorithms are K-nearest neighbor (KNN), support vector machine (SVM), decision tree (DT), logistic regression (LR), and multi-layer perceptron (MLP). To choose the best-performing machine learning algorithm in federated learning, the ADNI dataset has been used to compare the performance of different classifiers, as shown in Figure 1. In terms of total prediction accuracy, MLP outperforms all other models (indicated by * in Figure 1), with the greatest accuracy of 0.888889. This shows that MLP classifies occurrences with the fewest mistakes. Additionally, MLP performs well in terms of recall, accuracy, and F1 score. MLP demonstrates its capacity to accurately identify positive cases while maintaining a solid balance between recall and accuracy with recall and precision values of 0.794118 and 1.0, respectively, and an F1 score of 0.885246. MLP consistently beats other models regarding the accuracy, recall, precision, and F1 score compared to their performance. This suggests that MLP is more adept at detecting intricate patterns and asymmetrical connections in the data. Due to its multi-layer design, MLP can extract low-level and high-level information by learning hierarchical representations. By iteratively enhancing the model’s parameters, the gradient-based optimization methods used to train MLP, such as backpropagation, further improve the model’s performance. Based on the above discussion, MLP was chosen to be used in FL and subsequently implemented in hardware.

Each of the MLP’s three hidden levels includes 128 units, 64 units, and 32 units. These hidden layers are crucial, allowing the MLP to learn from the input data and provide meaningful representations. The entire learning process is conducted on one neural network, so the computational complexity is higher than that of the FL method. The activation function for all hidden layers has been determined to be the rectified linear unit (ReLU). ReLU introduces non-linearity into the model, allowing it to recognize intricate connections and patterns within the data. This activation function helps the model to more correctly represent the underlying structure of the input data by promoting the learning of hierarchical features. During training, 500 iterations is the maximum allowed. This option controls the number of times the MLP iterates over the training data. Applying an iteration constraint minimizes overfitting and compromises model performance and training duration. Deep learning typically uses the well-known optimization method, Adam, or Adaptive Moment Estimation. It combines the advantages of methods with a flexible learning rate with momentum-based approaches. The selection of four sites was made solely for illustrative reasons to show how well FL works in a dispersed environment. It was not meant to restrict the method’s adaptability or scope of use. As a matter of fact, the method easily supports more sites without compromising the model’s performance or changing the underlying algorithm. Regardless of the number of sites, the core ideas and procedures stay the same, making the system more flexible to a wider variety of real-world situations. Then, the hyperparameter of those respective models was shared with the central server to create a global model.

Our FL model is explained in Algorithm 1, which involves a server and multiple clients. The algorithm aims to train a global model by aggregating local updates from the clients [47]. The federated learning model is split between the server and client sides. The algorithm begins with the server initializing the global model $w_{0}$. Then, in each round of training, the server randomly selects a subset of clients, denoted by $S_{t}$, to participate in the training process. In parallel, each client *k* in $S_{t}$ performs a local update, denoted by the function ClientUpdate(k,w), using the current global model $w_{t}$. The local update is performed over *E* local epochs, where the client processes its local data in batches of size *B*. The client updates its local model for each batch using the learning rate η and the gradient ∇(w,b) computed from the batch. Once the local epochs are completed, the client returns its updated model *w* to the server.
**Algorithm 1:** Federated Learning [47].
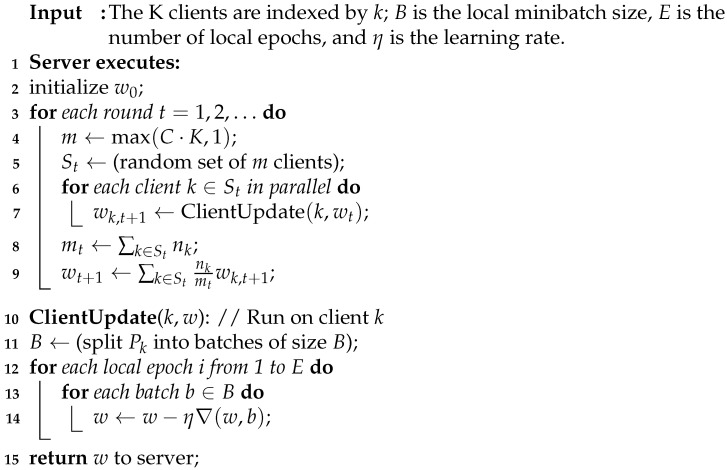


Back on the server, the algorithm aggregates the models received from the clients by computing a weighted average based on the number of samples $n_{k}$ each client has. The number of clients used in each round is determined by *m*, which is the maximum value between C·K and 1, where *C* is a constant. The resulting aggregated model is then used as the global model for the next round of training. The algorithm continues this process for multiple rounds, gradually improving the global model by incorporating local updates from different clients. The goal is to leverage the diversity of clients’ data while ensuring privacy and minimizing communication costs.

## 4. Implementation and Experimental Results

### 4.1. Block Diagrams

**Figure 2:**
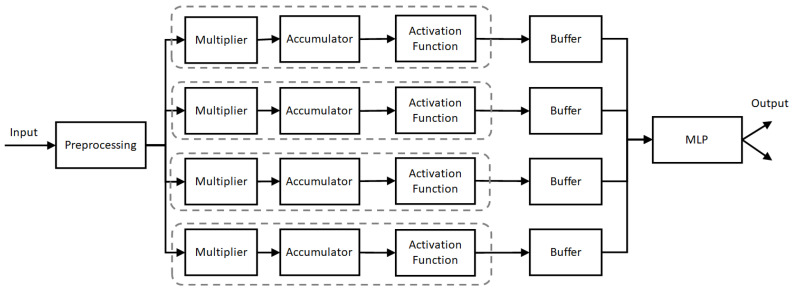
Block diagram of the FL method.

The FL method consists of multiple MLPs. Four MLPs are used for FL learning and one is used for the main learning, as shown in Figure 2. The first four MLPs are connected in parallel after the preprocessing stage. Using the Z-score method, the preprocessing stage performs standardization and normalization of the input data to make all the data values between 0 and 1. The FL has three stages: a multiplier, an accumulator, and an activation function. The multiplier is used to multiply the incoming data by the saved weight in memory. The multiplier uses 32 bits for the multiplication process. Each multiplication result is applied to the accumulator to add the new result to the previous accumulated value. The final value of the accumulator is applied to the activation function, which produces a high-output value when the input exceeds a threshold value and a low value when the input is lower than the threshold value. The output of each MLP is given by Equation (1).

(1) $$y_{i}=f\left(\sum_{m=0}^{K}W_{mi}*X_{m}+b_{i}\right)$$

where $X_{m}$ is the output of the *m*th node and *K* is the number of nodes, $W_{mi}$ is the weight from *m*th node to the *i*th node in the current layer. *f* is the activation function, and *b* is the used bias. The output of each MLP in FL is forwarded to a synchronization buffer. The buffer value is forwarded to the main MLP, which uses the average coefficient of the MLPs for the final classifications. Each MLP is designed using three hidden layers with 1500, 300, and 100 nodes, respectively.

### 4.2. Implementation and Experimental Results

**Figure 3:**
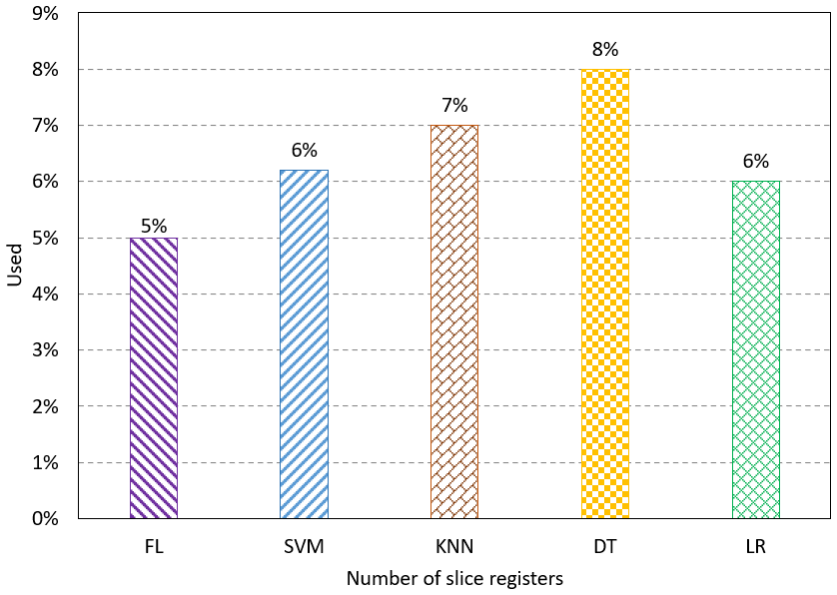
Resource utilization results for the number of slice registers.

**Figure 4:**
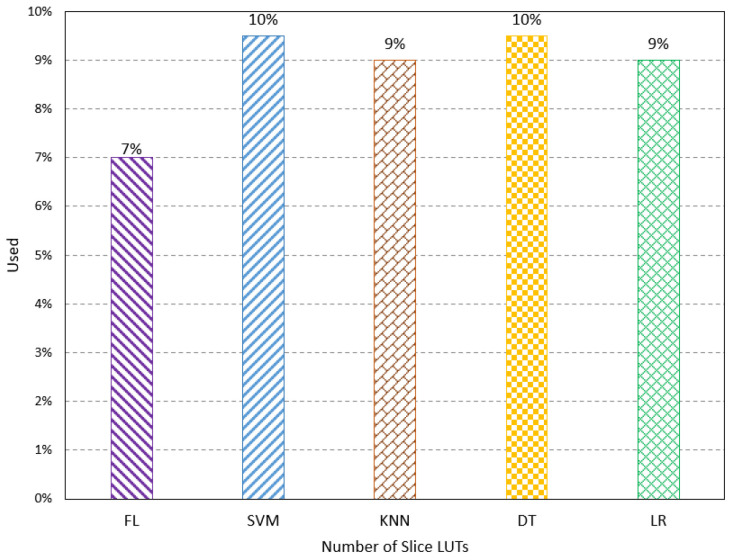
Resource utilization results for the number of slice LUTs.

The proposed method is designed using VHDL and implemented on FPGA Altera 10 GX, which has suitable resources for neural network computation and implementation. The proposed method’s resources, latency, and power consumption are studied. Resource utilization refers to the percentage of the resources used by the proposed method compared to the available resources on the FPGA. Some parameters are used to show the resources used, such as the number of slice registers, slice Look-Up Table (LUT), Buffers (BUFs), Digital Signal Processing (DSPs), Block RAM, LUTs, and Flip-Flop (FF). The number of slice registers indicates the number of registers mapped by the proposed method on the FPGA design. The registers handle the synchronization of the implementation that has different units working at different rates. Thus, it synchronizes the data movement between these units. The simulation result shows the FL method has a usage of 5%, which is lower than the other method, as shown in Figure 3. The number of slice LUTs shows the used LUTs of the proposed method to map functions of neural network computation. The results show the FL method has the lowest percentage of the used slice LUTs, as shown in Figure 4.

**Figure 5:**
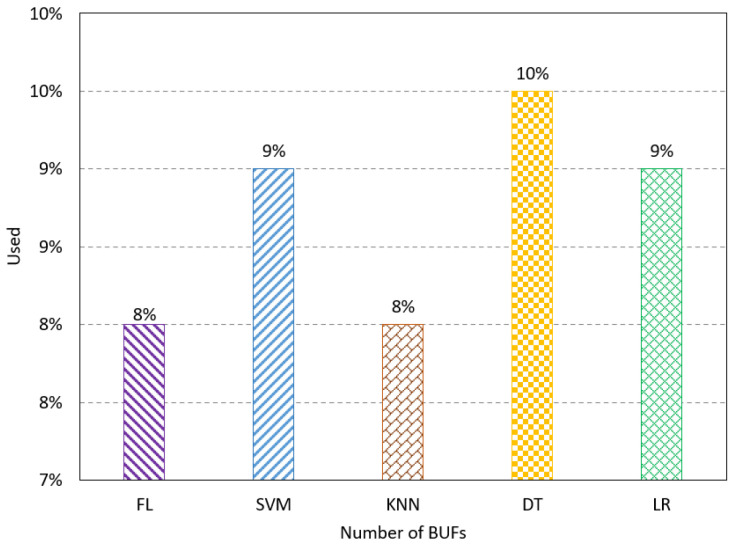
Resource utilization results for the number of BUFs.

**Figure 6:**
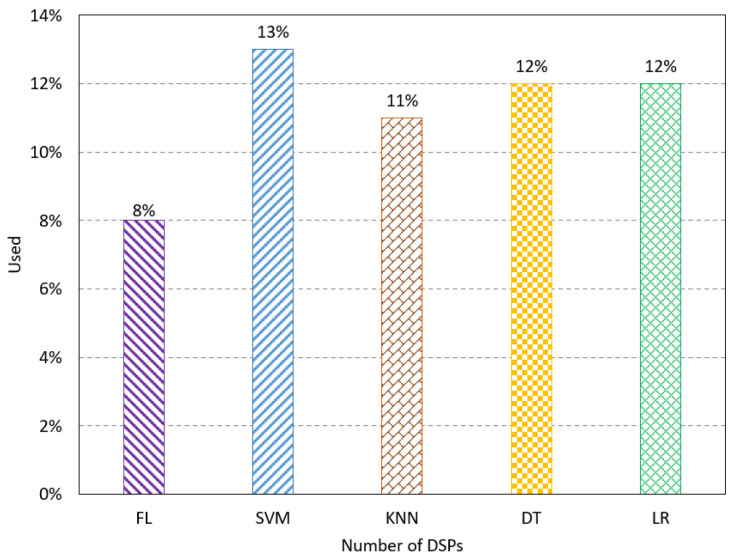
Resource utilization results for the number of DSPs.

**Figure 7:**
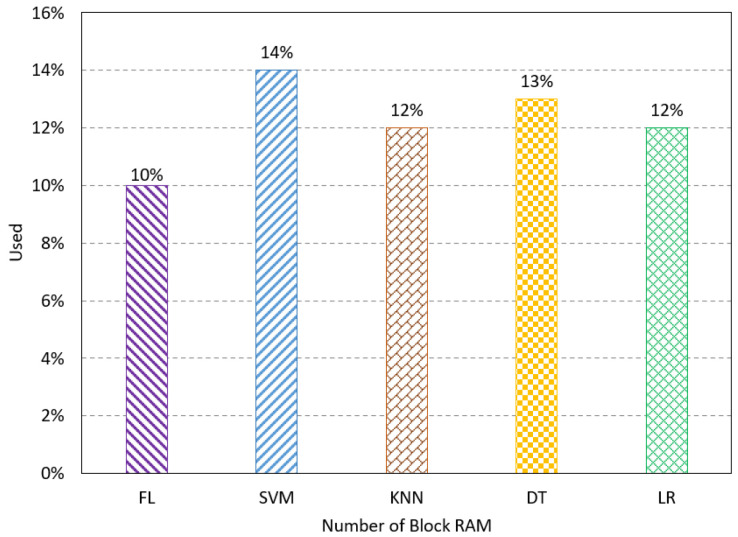
Resource utilization results for the number of RAM.

The number of BUFs refers to the buffers used for synchronization and isolation in the proposed design. The results show the FL method has a low resource utilization at 8%, as shown in Figure 5. The number of DSPs presents the number of signal processing units used for the computation. The FL method has the lowest percentage of DSPs used compared to the existing method, as shown in Figure 6. The number of block RAM indicates the used RAM unites on the FPGA to handle the memory transformation. The results show that the FL method uses 10% of the RAM units, which uses fewer resources than the existing methods, as shown in Figure 7.

**Figure 8:**
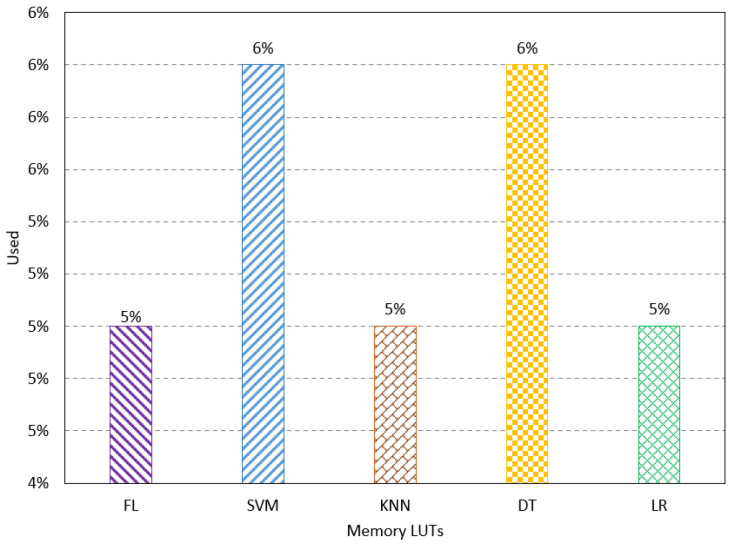
Resource utilization results for the number of memory LUTs.

**Figure 9:**
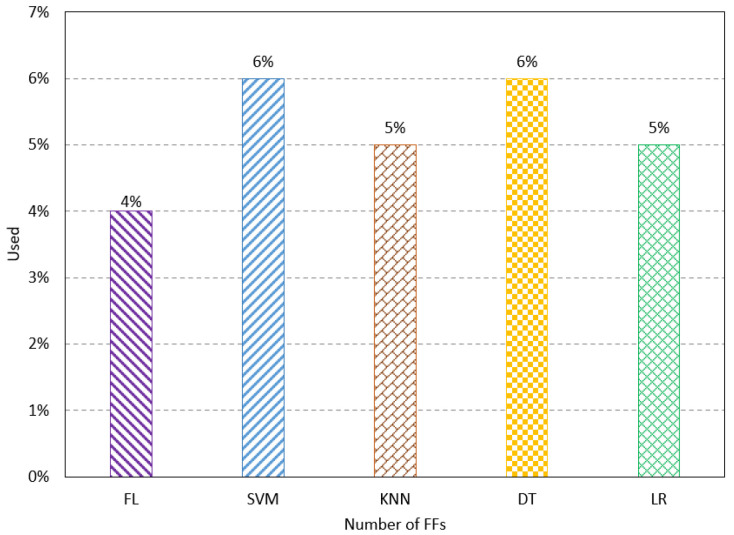
Resource utilization results for the number of FFs.

The same performance occurs with regard to the amount of memory used for LUTs, where the FL uses 5% of the available resources. In contrast, the other methods use more resources, as shown in Figure 8. The number of FFs presents the digital FFs used for connection and synchronization in the system. The results show the FL method has utilization of 4%, which is lower than that of the other methods, as shown in Figure 9. The overall results show that the FL method has a better performance in terms of resource utilization compared to the existing methods.

**Figure 10:**
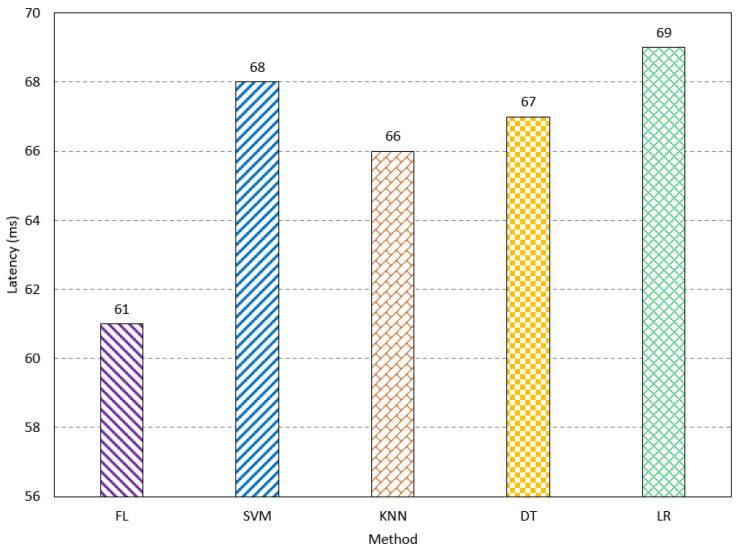
The latency performance of the FL method compared to the traditional methods.

**Figure 11:**
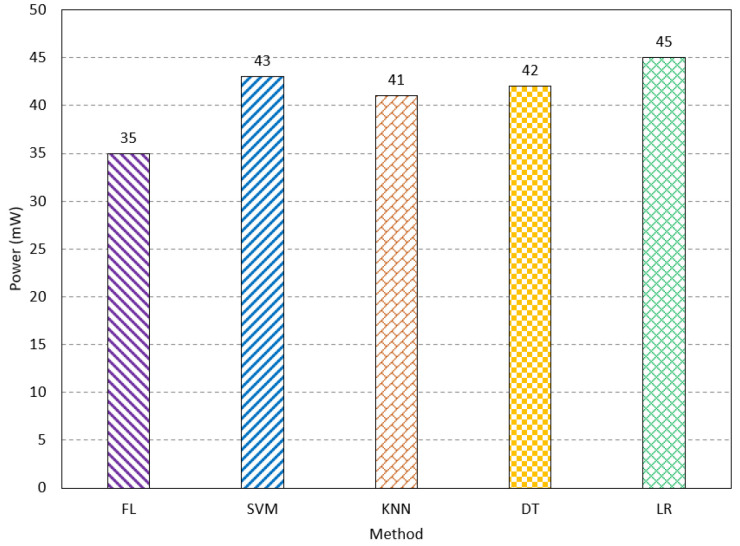
The processing power consumption of the FL method compared to the traditional methods.

**Figure 12:**
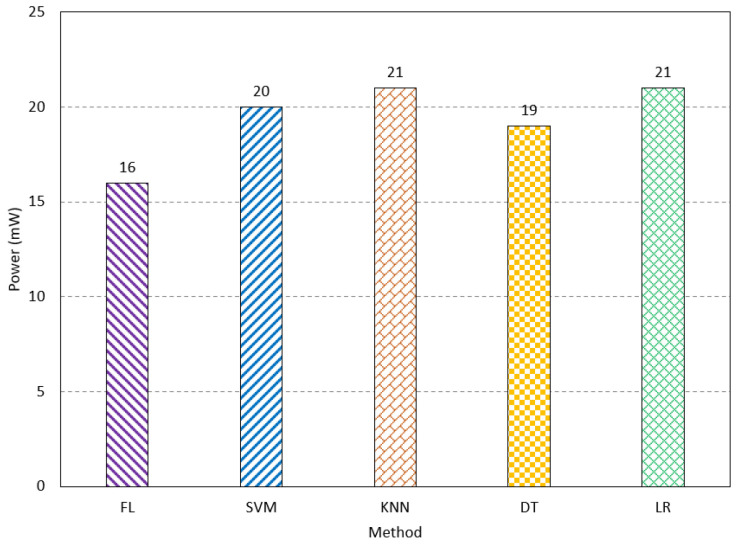
The communication power consumption of the FL method compared to the traditional methods.

The inference latency is studied for FL and traditional methods. The FL method has an inference latency of 61 ms. The SVM, KNN, DT, and LR have an inference latency of 68 ms, 66 ms, 67 ms, and 69 ms, respectively, as shown in Figure 10. This paper also studied both processing and communication power consumption. Processing power refers to the power consumed during the processing of the input features in learning and testing. The communication power indicates the consumed power of the network when sending and receiving the final result of the network. The FL method consumes the lowest processing power compared to the existing methods. The power consumption is measured using the Synopsys Design Compiler. It consumes 35 mw, while SVM, KNN, DT, and LR consume 43 mw, 41 mW, 42 mW, and 45 mW, respectively, as shown in Figure 11. The FL method has a communication power consumption of 16 mW, which is comparable to the other methods, as shown in Figure 12. The FL method has less processing and communication power due to the lightweight processing, as the data are divided into multiple networks. The results show the FL method is suitable for biomedical applications, especially for AD, as it has a low cost compared to the existing methods, while it has acceptable performance.

## 5. Conclusions

As the disease develops slowly over many years, Alzheimer’s disease (AD) profoundly affects patients and their families. Illness outcomes and consequences could be significantly affected if caught early and accurately. Blood samples are utilized in non-invasive medical testing to diagnose AD early. Using blood samples, this paper presented an FL-based diagnosis model for AD. We tested and compared the efficiency of our models using actual blood sample datasets obtained from the ADNI website. We employed a hardware acceleration approach to applying our FL model to speed up the training and testing procedures based on the massive data obtained for early AD detection. The proposed method uses VHDL hardware description language and an Altera 10 GX FPGA to study the hardware performance and cost. It achieved 35 mW power consumption for the processing phase and 19 mW power consumption for the communication phase. Additionally, the result shows it has 61 ms of inference latency. According to our performance outcome, our algorithms achieved an early detection accuracy that outperformed the existing algorithms while requiring fewer resources from the hardware point of view.

## Data Availability

https://adni.loni.usc.edu/data-samples/access-data/ (accessed on 5 August 2023).
